# Supplementary material for: Nucleoid-associated proteins shape the global protein occupancy and transcriptional landscape of a clinical isolate of Vibrio cholerae
Source: mSphere. 2024 Jun 26;9(7):e00011-24. doi: 10.1128/msphere.00011-24 (PMC11288032; doi:10.1128/msphere.00011-24)
Supplement: Supplemental material — Supplemental text, figures, and tables. [file msphere.00011-24-s0001.pdf]

Supplementary Material for “Nucleoid-associated proteins shape the global protein occupancy and transcriptional landscape of a clinical isolate of *Vibrio cholerae*”

Table of Contents

Supplementary Text **2**

Supplementary Figures **4**

Supplementary Figure1: 4
Supplementary Figure 2: 5
Supplementary Figure 3: 6
Supplementary Figure 4: 7
Supplementary figure 5: 8
Supplementary Figure 8: 11
Supplementary Figure 9: 12

Supplementary Tables: **13**

Supplementary Table 1: 13
Supplementary Table 2: 13

Supplementary Text

H-NS is likely causing negative occupancy signal in *V. cholerae* IPOD-HR data

While there is no large change in RNA polymerase occupancy in the *ΔtsrA,* and *Δihf* strains in the VPI-1 **(Figure 3C)**, the occupancy scores (IPOD and IPOD-HR rz-scores) are more negative for these strains compared to the *ΔvctA* control gene and the wild type strain. Although no large change in RNA polymerase occupancy is observed, *ΔtsrA* and *ΔihfA* strains still resulted in a more negative IPOD and IPOD-HR signal in this HAE compared to the wild type or the control gene deletion of VctA. This motivated us to assess the distribution of occupancy scores in the H-NS bound regions from a published study of V5-tagged H-NS ChIP-seq [71] from the strain of *V. cholerae* C6706 that we re-mapped to our wild type KDS1 reference genome **(Supplementary Figure 5B)**. The occupancy signals before and after RNA polymerase subtraction based on IPOD and IPOD-HR for the wild type strain are negative suggesting that H-NS may be a protein in our method where it produces negative signal due to its depletion from the interphase as was the case with Rok in *B. subtilis [21]*. We further assessed the distribution of occupancy scores at these regions in the *ΔihfA* and observe increased distribution of negative scores in the H-NS-bound regions from C6706, suggesting that the absence of IHF results, perhaps, in more H-NS binding, as it was demonstrated for the *tcpA* promoter in the absence of *ihfA* from cells grown in stationary phase in tryptic soy broth [40], or it allows the signal of H-NS occupied regions to be more negative due to lack of competition from other factors binding to the same regions. With this in mind we suggest that regions producing the negative occupancy signal may be due to H-NS.

This is again consistent with the interpretation that *V. cholerae* H-NS yields a negative IPOD signal due to depletion from the interface under the conditions used here; thus, lack of competition from other NAPs leads to an increased negative occupancy signal at H-NS bound regions due to reduced competition (either for binding or for interface partitioning), whereas deletion of H-NS leads to increased transcription of the same regions. However, we do not see large changes in RNA polymerase occupancy in the individual deletions of *tsrA* or *ihfA*, corroborating again that H-NS is still largely repressing CTX region **(Figure 3D, Supplementary Figure 4, 5C)**. In future studies, it will be important to demonstrate what constitutes the negative occupancy during IPOD-HR in *V. cholerae*, whether it is mainly H-NS or a mixture of proteins and why the negative occupancy signal appears strongly when proteins known to act at

those loci such as TsrA and IHF are absent. A similar question remains unanswered for the SOS-induced effect on protein occupancy, where some regions produce more negative occupancy signals than others, such as the PLE **(Figure 5C)**, perhaps suggesting the binding of H-NS or other protein factors to this region during DNA damage.

Supplementary Figures

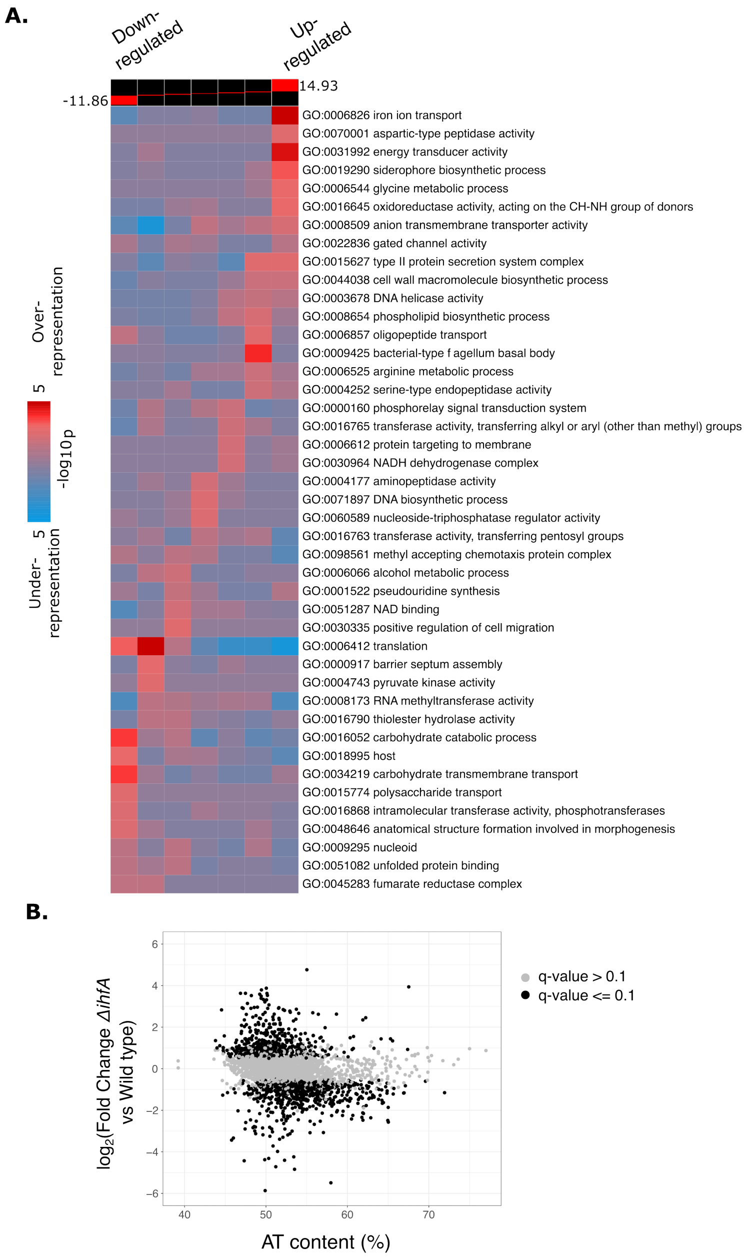

Figure S1:

(A) GO term enrichment classification analysis of RNA-sequencing results for Δ*ihfA* cells relative to WT. Within each of seven equally-populated bins discretizing the $\log_2$ fold change, the heat map shows the enrichment or depletion of members of the indicated GO term in that expression bin.

(B) AT percentage of differentially expressed genes in Δ*ihfA* versus wild type.

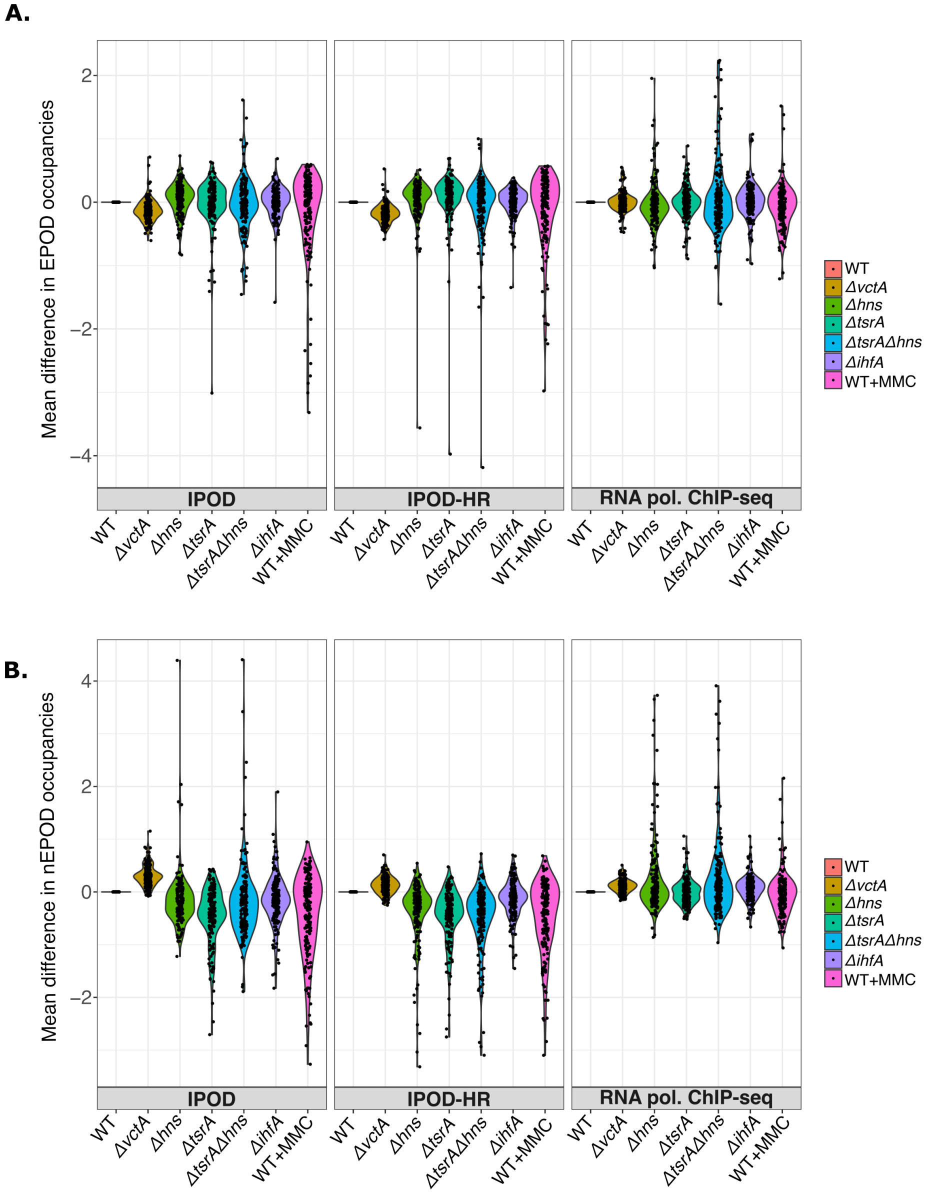

Figure S2:

A) Mean differences in protein occupancy of EPODs between the average of indicated genotypes and the wild type. Values are plotted separately for IPOD, IPOD-HR and RNA polymerase ChIP-Seq.
B) Mean differences in protein occupancy of negative EPODs (nEPODs) between the average of indicated genotypes and the wild type. Values are plotted separately for IPOD, IPOD-HR and RNA polymerase ChIP-Seq.

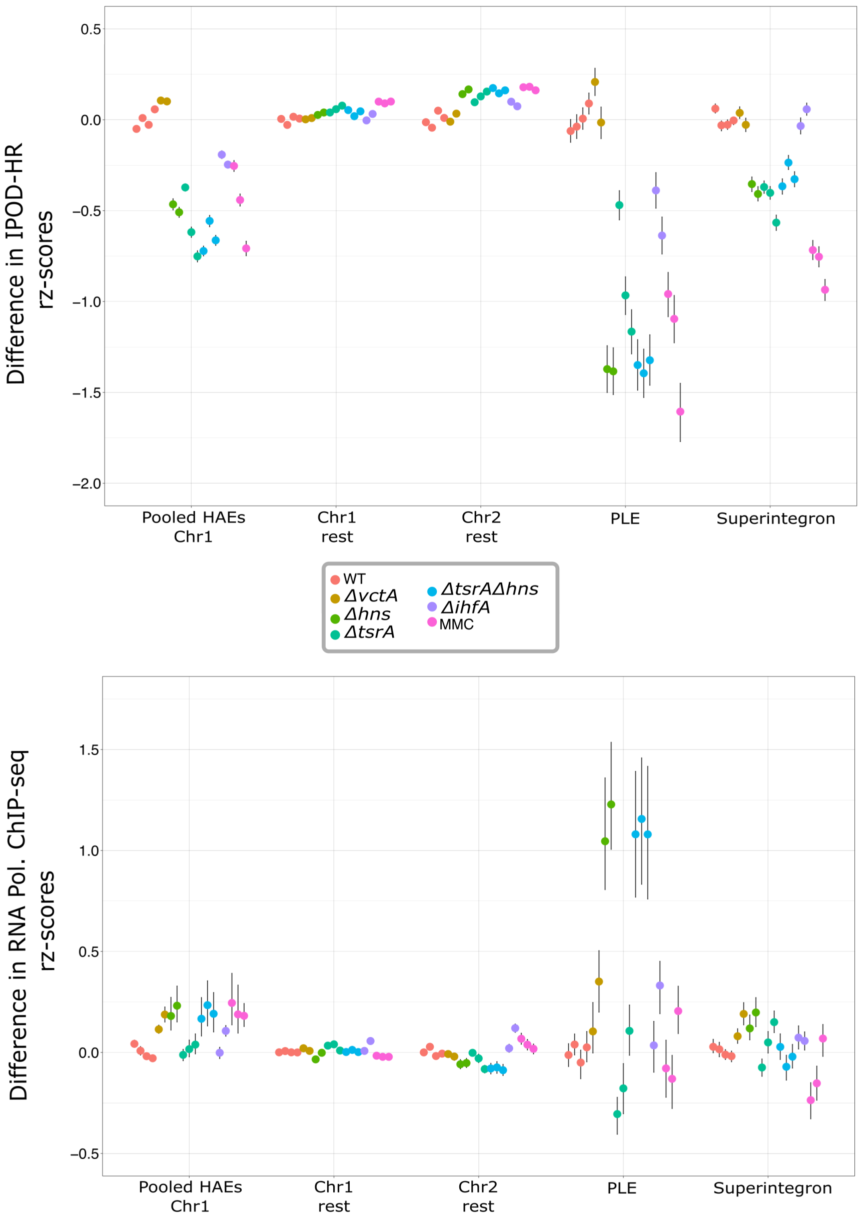

Figure S3:

Difference (relative to WT) in IPOD-HR occupancies and difference (relative to WT) in RNA polymerase ChIP-seq rz-scores in indicated genotypes at individual replicate levels. For IPOD-HR, a 50 bp rolling median was used as the fundamental unit of data centered at each genomic location, and the plotted values reflect the pseudomedian of those values across the indicated genomic features. Error bars show 95% confidence intervals calculated using the wilcox.test function in. For RNA polymerase ChIP-seq, we followed a similar procedure as for IPOD-HR, except that we used gene-level means of the ChIP occupancies as individual units of data for the pseudomedian calculations.

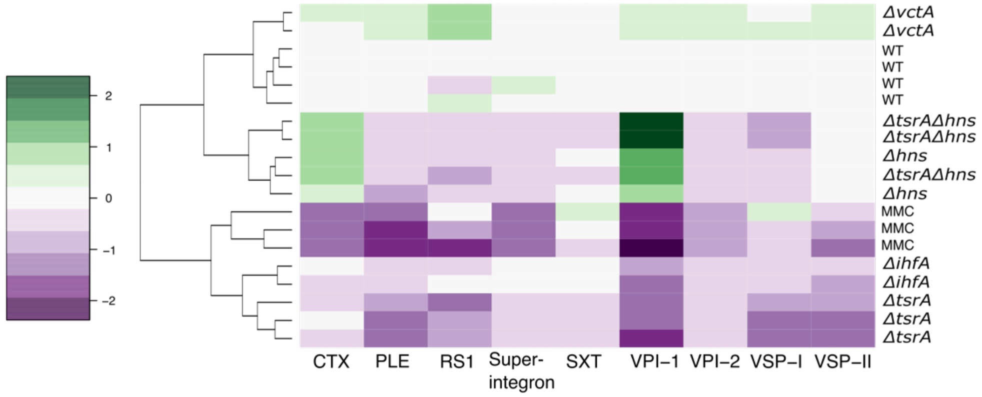

B.

Differences in IPOD-HR rz-scores

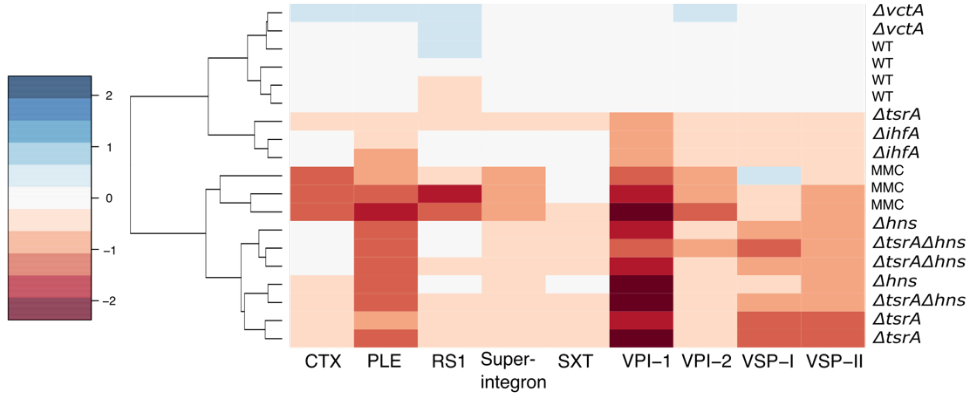

C.

Differences in RNA Pol. ChIP-seq rz-scores

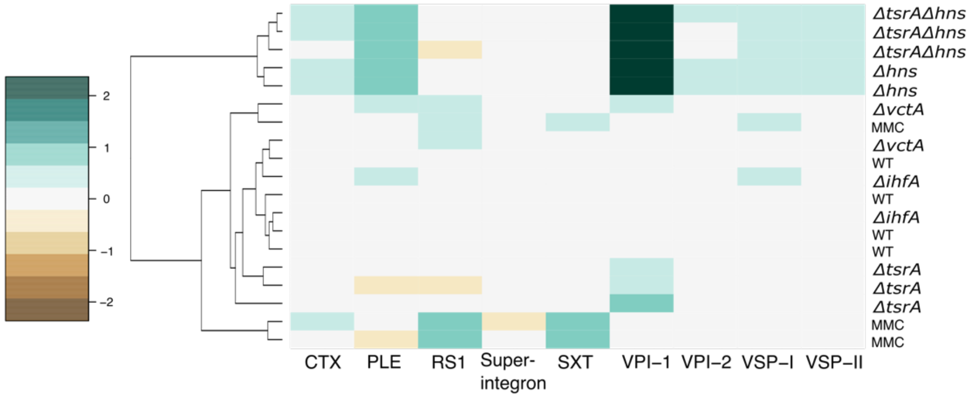

Figure S4:

Heatmaps showing the differences of means of A) IPOD, B) IPOD-HR and C) RNA polymerase ChIP-seq rz-scores in the indicated conditions at the biological replicate level, relative to the average for wild type.

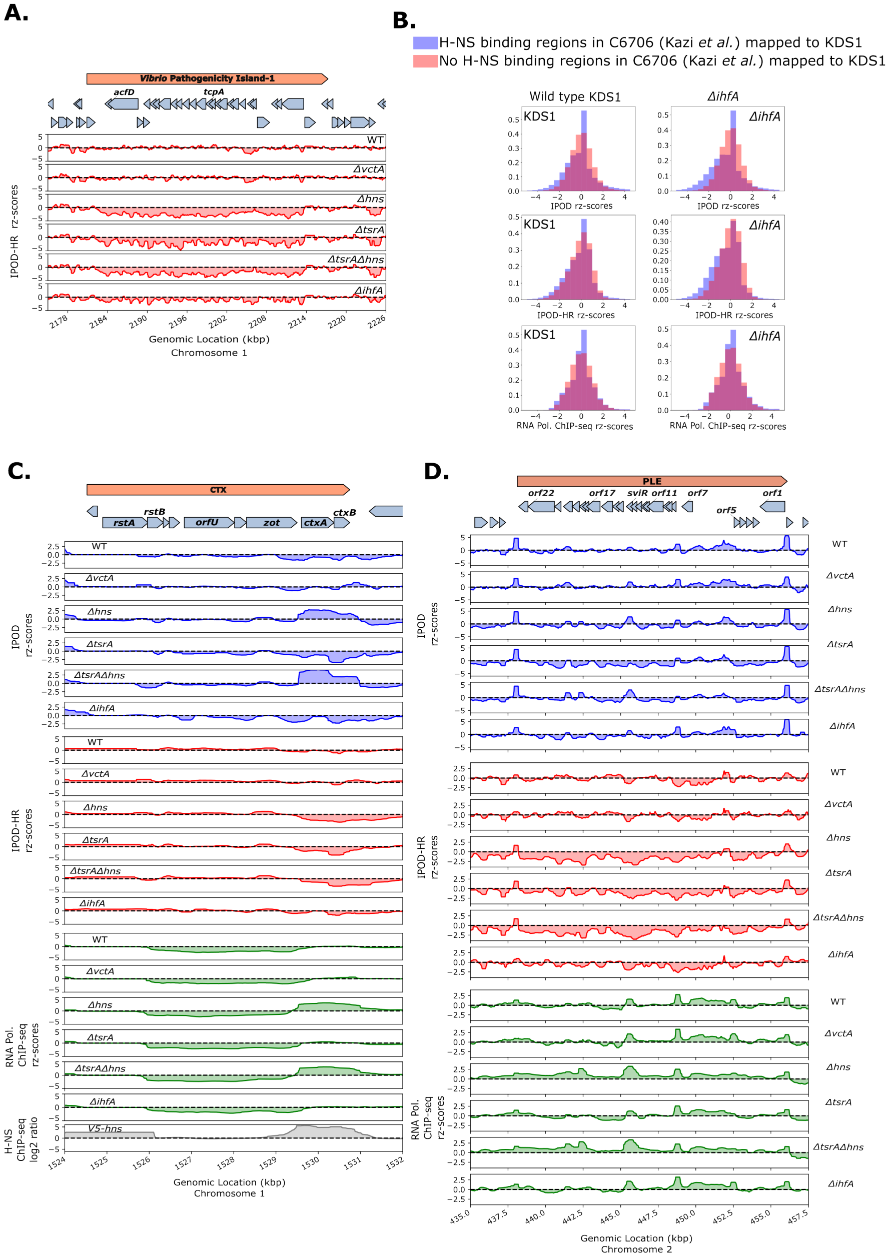

Figure S5:

A) IPOD-HR occupancy traces in the VPI-1 island for the indicated strains.
B) Distribution of scores in the wild type and Δ*ihfA* Vibrio cholerae KDS1 in the regions obtained from V5-H-NS ChIP-seq study in strain C6706 (data from [71]) in the main text).
C) IPOD, IPOD-HR and RNA polymerase ChIP-seq occupancy tracks for all of the genotypes in the CTX region of *V. cholerae,* with the gray track being the V5-H-NS ChIP-seq from C6706 (data from [71]).
D) IPOD, IPOD-HR and RNA polymerase ChIP-seq occupancy tracks for all of the genotypes in the PLE region of *V. cholerae.*

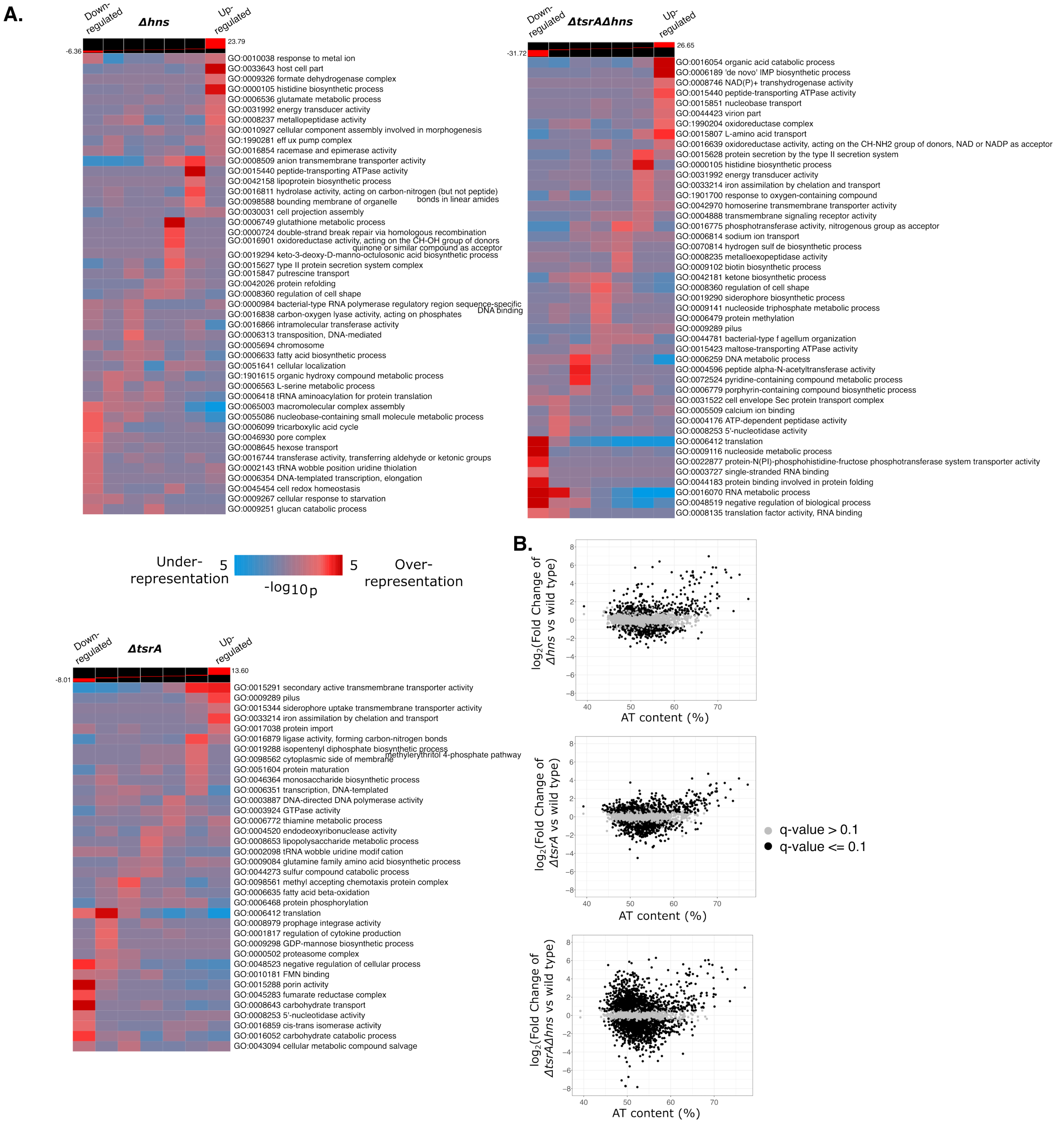

Figure S6:

A) GO term enrichment classification analysis of RNA-sequencing results of indicated genotypes: Δ*hns,* Δ*tsrA,* and Δ*hns*Δ*tsrA.* For each case, the heatmap shows a division of genes into seven equally-populated bins discretizing the $\log_2$ fold change, the heat map shows the enrichment or depletion of members of the indicated GO term in that expression bin.

B) AT percentage of differentially expressed genes in Δ*hns,* Δ*tsrA,* and Δ*hns*Δ*tsrA* versus wild type. Significant differentially expressed genes (q-value less than or equal to 0.1) are indicated in black and q-value of greater than 0.1 are indicated in gray.

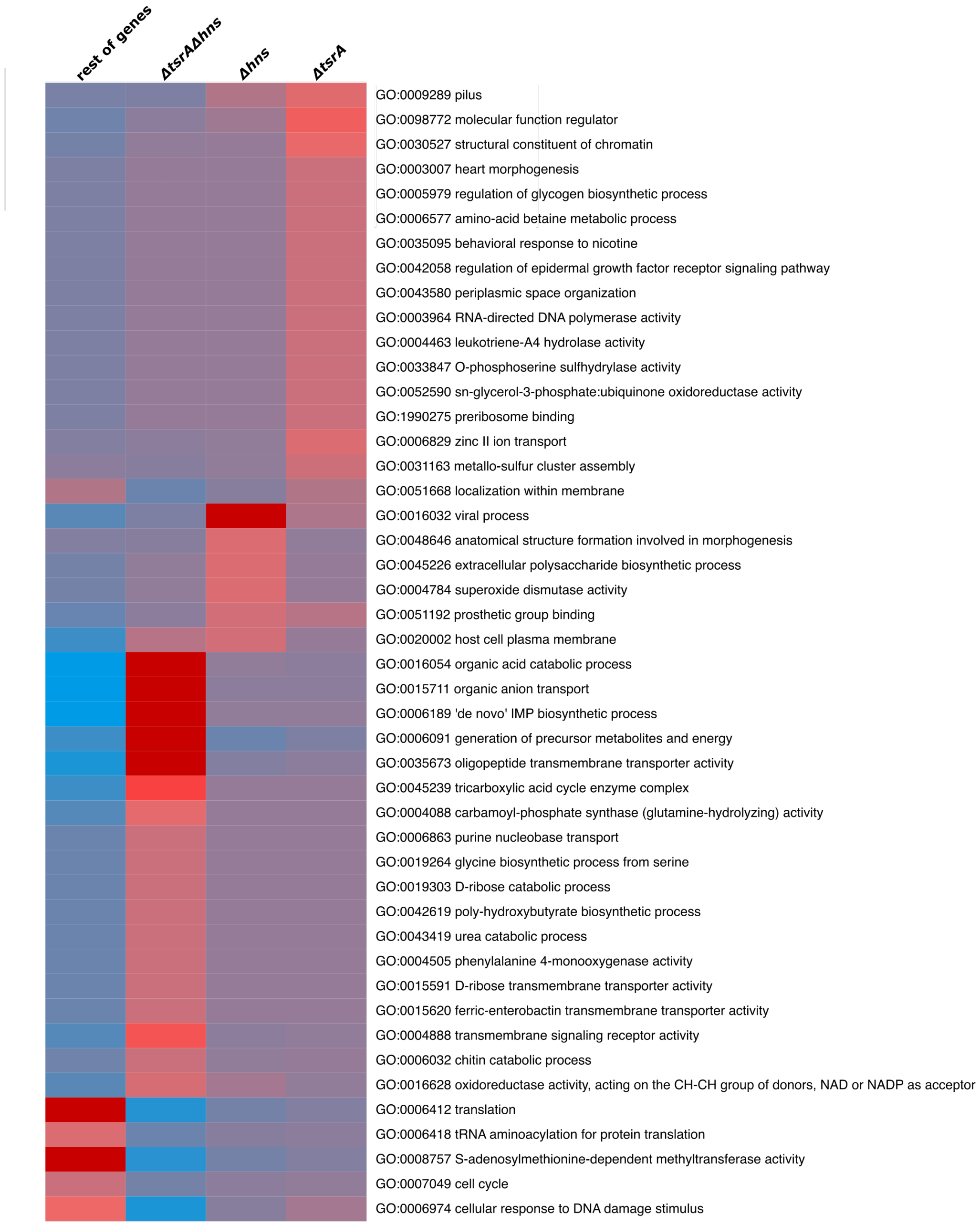

Figure S7:

GO term enrichment classification analysis of the upregulated genes (defined by a q value <0.1 and a $\log_2$ fold change at least three times the standard error of the mean) from the RNA-sequencing results of indicated genotypes: Δ*hns,* Δ*tsrA,* and Δ*hns*Δ*tsrA.*

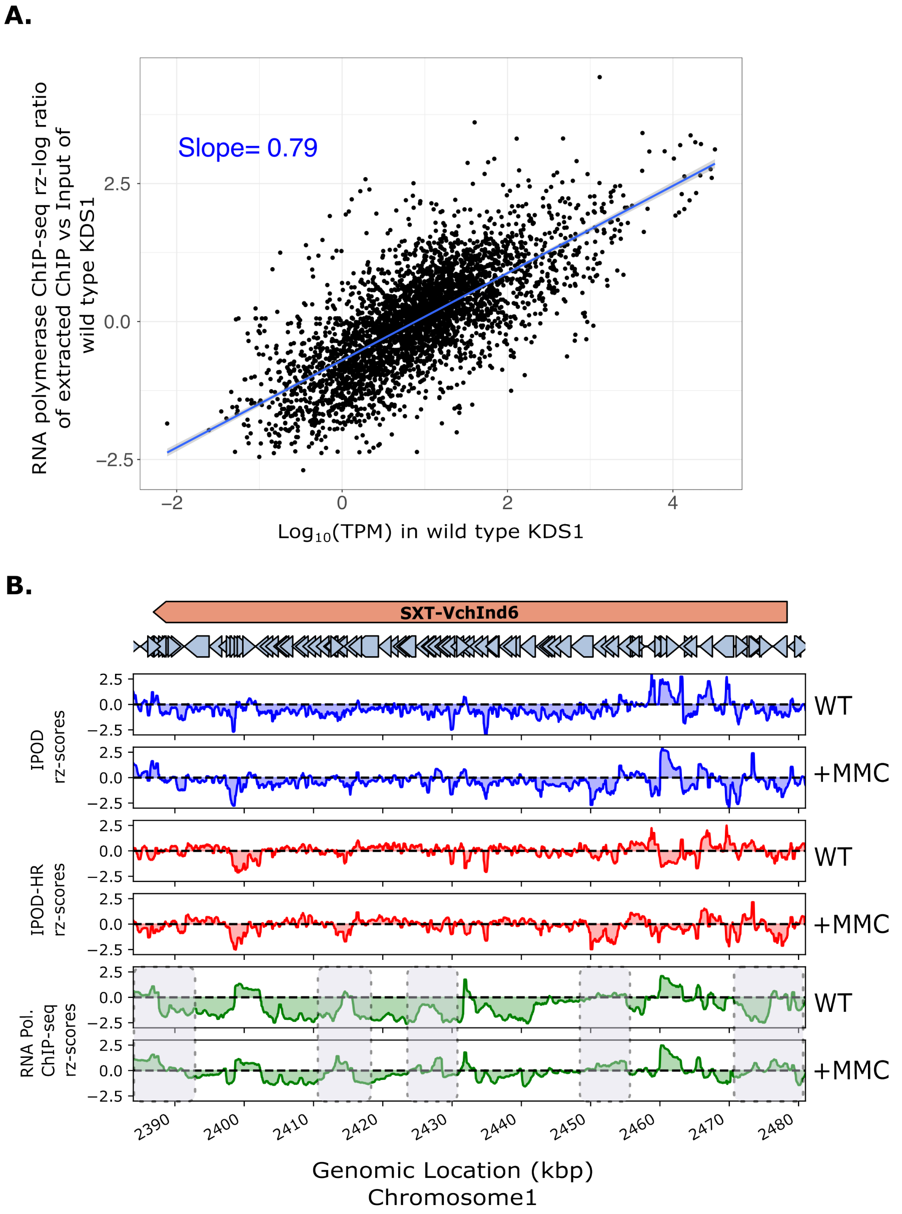

Figure S8:

A) Correlation plot of rz-log ratios of RNA polymerase ChIP-seq vs. Input (y axis) with RNA-seq log10(Transcripts per million (TPM)) (x axis) in the wild type KDS1. The line represents a robust linear model of Log10(TPM) as a function of RNA polymerase ChIP-seq in wild type. The slope is shown in blue. Genes with less than 0.0001 TPM are omitted from the plot.
B) Occupancy traces of IPOD, IPOD-HR and RNA polymerase ChIP-seq of the whole SXT-VchInd6 genomic feature in untreated and MMC treated wild type *V. cholerae*. The shaded rectangles are shown for visual comparison of regions with differences in RNA polymerase binding between the untreated and MMC treated cells.

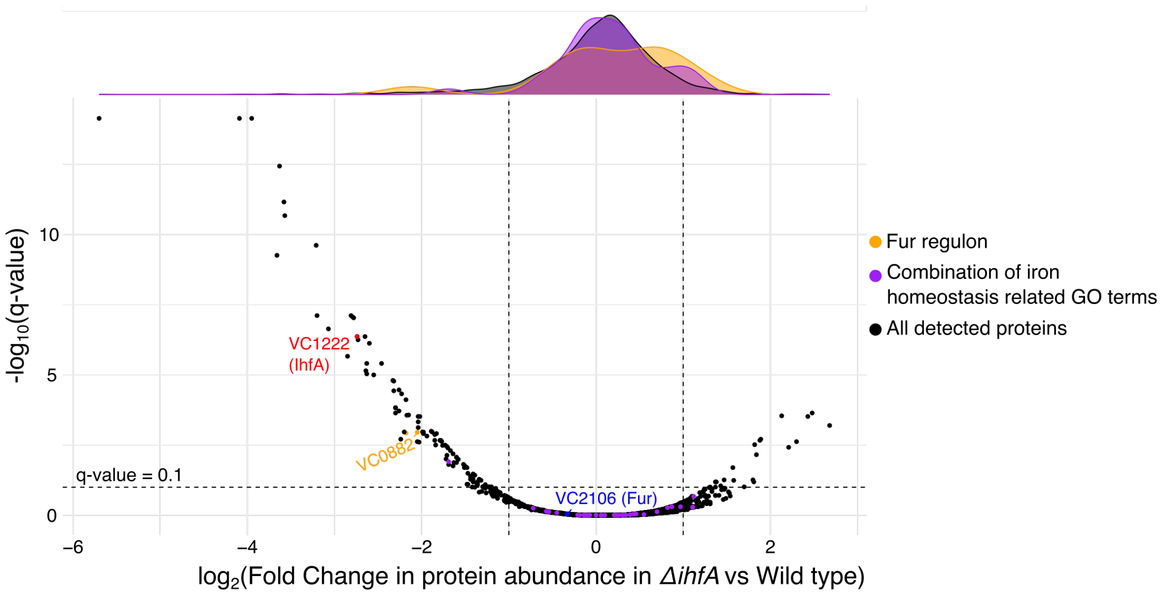

Figure S9:

Volcano plot of differential protein abundances obtained from Tandem Mass Tag Mass Spectrometry from the strains lacking *ihfA* vs. wild type. Fur regulon and iron homeostasis protein groups are color-matched to the RNA-seq volcano plot for consistency (Figure 2B). The horizontal dashed line indicates the $-\log_{10}$(q-value) of 0.1 and the left and right vertical dashed lines represent log2(fold change) of -1 and 1, respectively.

Supplementary Tables:

Table S1: Numbers of overlapping and unique IPOD-HR peaks in the conditions tested compared to the 122 peaks in the wild type.

Strain	Number of peaks	Overlapping peaks	Unique peaks
Δ*ihf*	106	38	68
Δ*hns*	100	52	48
Δ*tsrA*	98	54	44
Δ*vctA*	116	64	52
Δ*tsrA*Δ*hns*	121	42	79
MMC	33	24	9

Table S2: Bacterial strains used in this study.

Strain and genotype	Source	Lab identifier
V. cholerae PLE(+)	Seed et al., 2013 [55]	KDS1
V. cholerae PLE(+)Δ*hns*::frt-spec-frt	This study	DD427
V. cholerae PLE(+)Δ*ihfA*	This study	DD430
V. cholerae PLE(+)Δ*tsrA*::frt-kan-frt	This study	DD601
V. cholerae PLE(+)Δ*tsrA*::frt-kan-frt + Δ*hns*::frt-spec-frt	This study	DD607
V. cholerae PLE(+)Δ*vctA*	This study	KS2599
